# “Management of myositis associated interstitial lung disease”

**DOI:** 10.1007/s00296-023-05336-z

**Published:** 2023-05-01

**Authors:** Lorraine Thong, Liam J. Chawke, Grainne Murphy, Michael T. Henry

**Affiliations:** 1 Department of Clinical Medicine, Trinity Translational Medical Institute, Trinity College Dublin, St. James Hospital, Dublin, Ireland; 2 Department of Clinical Medicine, University Hospital Kerry, Kerry, Ireland; 3 Department of Rheumatology, Cork University Hospital, Cork, Ireland; 4 Department of Respiratory Medicine, Cork University Hospital, Cork, Ireland

**Keywords:** Idiopathic inflammatory myopathies, Myositis, RP-ILD, M-ILD, Interstitial lung disease, Management, Treatment

## Abstract

Idiopathic inflammatory myopathies (IIM) are rare disorders characterised by the presence of skeletal muscle inflammation, with interstitial lung disease (ILD) being the most frequent pulmonary manifestation. The spectrum of clinical presentations of myositis related ILD (M-ILD) encompasses a chronic process to a rapidly progressive ILD (RP-ILD); which is associated with a high mortality rate. The most effective treatments remain controversial and poses a unique challenge to both rheumatologists and respiratory physicians to manage. Given the rare heterogenous nature of M-ILD, there is a paucity of data to guide treatment. The cornerstone of existing treatments encompasses combinations of immunosuppressive therapies, as well as non-pharmacological therapies. In this review, we aim to summarize the current pharmacological therapies (including its dosing regimens and side effects profiles) and non-pharmacological therapies. Based on the existing literature to date, we propose a treatment algorithm for both chronic M-ILD and RP-ILD.

## Introduction

Idiopathic inflammatory myopathies (IIM) are a heterogenous group of disorders encompassing polymyositis (PM), dermatomyositis (DM), clinically amyopathic dermatomyositis (CADM), and immune mediated necrotising myopathies (IMNM) [1]. Considered a rare disorder, the prevalence of IIM ranges from 2.4 to 33.8 per 100,000 population and incidence of 1.16–19 per million/year [2]. The subgroup classification criteria have evolved overtime, from Peter and Bohan in 1975, to the current classification criteria by the European League Against Rheumatism (EULAR) [3–5].

IIM has a wide range of extra-muscular manifestations, with interstitial lung disease (ILD) being the most common with a global prevalence rate of approximately 41% among IIM patients [6, 7]. Despite ILD in IIM being associated with a high mortality rate, it is not included the latest classification criteria for IIM [5]. More recently, the British Society of Rheumatology published a guideline on management of paediatric, adolescent, and adult patients with IIM including myositis associated ILD (M-ILD) [8]. However, recommendations for the treatment of M-ILD are mostly conditional and based on low level of evidence. Furthermore, there has since been the release of a sub analysis of the RECITAL trial involving patients with M-ILD. To our knowledge, this is the first randomized and blinded study looking at the effectiveness of immunosuppression in this cohort of patients. Certainly, the lack of high-quality evidence for the treatment of M-ILD is lacking and worrying given the high disease burden of M-ILD and each treatment option poses a unique challenge to both rheumatologists and respiratory physicians. In this literature review, we summarize the current pharmacological and non-pharmacological therapies available for the treatment of M-ILD and propose a treatment algorithm for both chronic M-ILD and RP-ILD to ease clinician decision making when treating this disease.

### Search ﻿strategy

A search strategy for literature was adopted as described by Gasparyan AY et al. [9]. To ensure a thorough search and adequate relevant information was obtained, we searched MEDLINE/PubMed and SCOPUS data bases. As we felt that the subject of our review was niche and expected the evidence at present to be limited, we did not set a time frame restriction initially. Keywords used include “myositis AND interstitial lung disease AND (treatment OR management)”, “idiopathic inflammatory myopathies AND interstitial lung disease AND (treatment OR management)”, “rapidly progressive interstitial lung disease AND (treatment OR management)”, “chronic interstitial lung disease AND (treatment OR management)”, and “myositis associated interstitial lung disease AND (treatment OR management)”. Thereafter, duplicates and irrelevant articles were identified and removed. All randomized controlled trials, observational studies, and retrospective studies were included. Consideration was given to case reports and case series. Research articles and reviews were also considered for discussion points.

### D﻿iagnostic value of myositis specific antibodies (MSA)

The diagnosis of IIM involves serological testing for the presence of myositis specific antibodies (MSA), skeletal muscle biopsy, and MRI imaging of affected muscle compartment(s) [10]. MSA are diagnostically essential in allowing the differentiation of the various myositis phenotypes. Furthermore, the discovery of MSA has led to a reduction in diagnostic delays, avoidance of unnecessary investigations, and facilitated a more personalised approach to the management of these condition [11]. While MSA are highly specific for IIM, clinicians should refrain from fully relying on MSA for diagnostic purposes due to its low positive predictive value [5, 12]. Furthermore, as MSA are more widely used as part of ILD work-up or diagnosis (outside a well-defined cohort of IIM patients), this will invariably lead to a low pre-test probability [12].

The more commonly detected MSA implicated in ILD, are the antibodies directed against aminoacyl-tRNA synthetase enzymes (ARS antibodies: anti-Jo-1, anti-PL7, anti-PL12, anti-EJ, anti-OJ, anti-KU) and is now distinctively known as Anti-synthetase Syndrome (ASS) [13]. Less commonly, anti-melanoma differentiation association gene-5 (MDA5) autoantibodies have also been observed to be linked with ILD [13]. While Myositis Associated Antibodies (MAA) such as anti PM-SCL may have a diagnostic role in IMM, they are less specific and may be elevated in other rheumatological conditions such as scleroderma [14] (Table 1).

**Table 1:** Myositis specific and associated autoantibodies

Myositis specific antibody	Cutaneous and musculoskeletal features	Other associated features
ARS antibodies: Anti-Jo-1, PL7, PL12, EJ, OJ, KS, Hu, Zo	Anti-synthetase syndrome: Myositis, mechanics hand, fever, inflammatory arthritis, Raynaud’s phenomenon	ILD
Anti-Mi-2α/ Mi-2β	Mild myositis, cutaneous dermatomyositis	Low risk of ILD and malignancy
Anti-SRP	Necrotising myositis	Cardiovascular involvement
Anti-MDA5	CADM, Mucocutaneous ulceration,	RP-ILD, vasculopathy
TIF1 gamma / NXP-2	Dermatomyositis	Increased risk of malignancy
Anti-HMGCR	Statin-induced necrotizing myopathy	Dysphagia

Myositis associated antibody	Cutaneous and musculoskeletal features	Other associated features
Anti PM-SCL 100/Anti PM-SCL 75	Raynaud phenomenon, calcinosis	GERD
Anti-Ku	Myositis	GERD, serositis
Anti-Ro (SSA)/ Anti-La (SSB)	Sjogren’s syndrome	Auto-immune congenital heart block
Anti-U1RNP	Mixed connective tissue disease, SLE	ILD, pericarditis

*ARS* aminoacyl-tRNA synthetase enzymes; *CADM* Clinically amyopathic dermatomyositis; *HMGCR* HMG-CoA reductase; *ILD* Interstitial lung disease; *MDA5* melanoma differentiation associated gene 5; *NXP-2* nuclear matrix protein 2; *RP-ILD* Rapidly progressive interstitial lung disease; *SRP* signal recognition particle; *TIF* transcriptional intermediary factor

### Interstitial lung disease, a challenging extra-muscular manifestation of IIM

Counterintuitively, in patients with IIM, ILD may precede muscular symptoms in about 20% of cases and factors such as older age at presentation, lower forced vital capacity (FVC), high serum ferritin levels, and presence of anti-MDA5 autoantibodies, all lead to less favourable outcomes [15].

The initial presentation of a (M-ILD) usually follows an insidious course, and in some cases, patients are asymptomatic with the ILD being an incidental radiological finding [16]. Others may present with respiratory symptoms and muscle involvement [16]. Heterogeneity in initial presentation and clinical course is attributed and dependent on the various MSA and MAA that patients may have.

High resolution CT (HRCT) is the gold standard for the diagnosis of ILD. Radiologically, the most common features detected are ground glass opacites, bilateral reticulations, and traction bronchiectasis [17]. The most common radiological pattern seen in these patients are non-specific interstitial pneumonia (NSIP) and organizing pneumonia (OP), although, mixed NSIP-OP and usual interstitial pneumonia (UIP) patterns have also been reported [17, 18] (Table 2). Lung biopsy is rarely required, as the diagnosis can be adequately made with clinical history, MSA status, and radiological imaging. ILD, where it is present in about a third of these patients, a significant proportion of these develop into rapidly progressive ILD (RP-ILD) [19–21].

**Table 2:** Typical High-resolution computed tomography patterns seen in myositis ILD. Modified from the ATS/ERS International Multidisciplinary Consensus Classification of the Idiopathic Interstitial Pneumonias [22]

Radiological pattern	NSIP	OP	UIP
HRCT features	Lower lobe predominant (often with lower lobe volume loss	Typical basal predominance	Lower lobe predominant
	Subpleural sparing (around 50%)	Subpleural, perilobular, and/or peribronchovascular distribution	Subpleural reticulation
	GGO and reticular abnormality	Patchy air space consolidation/nodules with air bronchograms	Honeycombing
	Traction bronchiectasis	Patchy GGO	Traction bronchiectasis

*ATS* American Thoracic Society; *ERS* European Respiratory Society; *GGO* Ground glass opacification; *HRCT* High resolution computed tomography; *NSIP *Non-specific interstitial pneumonia; *OP* Organizing pneumonia; *UIP* Usual interstitial pneumonia

### Rapidly progressive interstitial lung disease

At present, there is no formal definition for myositis RP-ILD, although, experts concur that the progression of ILD is acute, occurring over a period of weeks to months [23–25]. Various criteria have been proposed to clearly differentiate RP-ILD from chronic M-ILD; some experts suggest worsening symptoms (hypoxaemia and dyspnoea) on top of worsening of fibrosis on HRCT (> 10% increase of the HRCT score) and/or reduction of absolute of FVC by > 10% [24, 25]. Furthermore, the presence of Anti-MDA5 autoantibodies are highly associated with RP-ILD with this association first established in 2005 by Sato et al. Additionally, RP-ILD has a higher prevalence in the Asian population suggesting possible genetic and environmental predispositions [6, 15]. The clinical phenotype often associated with anti-MDA5 RP-ILD is CADM, where there is absence of muscle involvement, presence of specific cutaneous manifestations such as Gottron’s ulcers/papules, and polyarthritis. Crucially, RP-ILD may precede cutaneous manifestations and is associated with a poorer prognosis [26–28].

Diagnostic work up for suspected cases include a myositis panel (including anti-MDA5), creatine kinase, and/or aldolase, an MRI to facilitate a targeted muscle biopsy, and ferritin levels (> 1500 ng/ml infers a poorer prognosis) [29]. Radiological clues that point towards RP-ILD is the presence of basal consolidations and diffuse ground grand opacities, with possible background changes of chronic/slowly progressing M-ILD [30, 31].

### Pharmacological management

Presently, the treatment for chronic M-ILD and RP-ILD is challenging as there are limited number of clinical trials hence, lack of evidence-based treatment guidelines. This is particularly worrying for cases of RP-ILD where it is associated with a high mortality rate. In this section we will discuss various agents of interest and provide a summary of the current level of evidence, doses, and recommended monitoring (Table 3).

**Table 3:** Doses and monitoring of therapeutic agents in the treatment of M-ILD

Medication	Highest level of evidence	Recommended use	Dose	Recommended monitoring
Glucocorticoids	Meta-analysis of mostly retrospective studies [33]	Initial treatment	PO prednisolone 0.5–1 mg/kg/day [34]	Annual densitometry
			IV methylprednisolone 1 g/day for 3 days [35]	Glucose monitoring
Azathioprine	Retrospective studies [39, 40]	First line steroid sparing agent for M-ILD	2 mg/kg/day PO [39]	FBC and LFT every 2 weeks for 4 weeks, then consider monthly once stable
		Add on treatment for RP-ILD		Sunscreen
Mycophenolate Mofetil	Retrospective studies [39, 42]	First line steroid sparing agent for M-ILD	Up-titrate to 2 g/day PO	FBC, LFTS’s every 2 weeks, then consider monthly once stable
		Add on treatment for RP-ILD	Max 3 gms daily [39, 42]	
Rituximab	Phase IIb randomized, double blind, superiority trial [49]	First line for RP-ILD	1000 mg IV D1 and D14 [49]	CD19/20 levels
		Add on treatment for M-ILD		Hepatitis and latent TB screen (QuantiFERON® assay)
Cyclophosphamide	Phase IIb randomized, double blind, trial [49]	First line for RP-ILD	600 mg/m2 body surface area every 4 weeks IV for six doses [49]	FBC every 2 weeks initially
		Add on treatment for M-ILD		
Tacrolimus	Randomized, open-label comparative trial [61]	Additional treatment option	0.075 mg/kg PO to achieve trough level of 5–20 ng/ml [54, 61]	FBC, renal profile, drug trough level
Cyclosporin A	Randomized, open-label comparative trial [61]	Additional treatment option	4 mg/kg/day PO, aim for peak levels of 1000 ng/ml [60] or trough level of 100–150 ng/mL [61]	FBC, LFT, renal profile, blood pressure monitoring
Tofacitinib	Small open label prospective trial [70]	Additional treatment option	5 mg PO twice daily [70]	Monitor for signs of infections
Nintedanib	Sub-analysis of the INBUILD trial [62]	Additional treatment option	150 mg twice a day [62]	Monitor LFT
Pirfenidone	Small open-label trial [63]	Additional treatment option	1800 mg daily [63]	Monitor LFT
				Sunscreen
IVIG	Retrospective study [73]	Salvage therapy	400 mg/kg/day IV × 5 consecutive days per month (period of 6 months) [73]	FBC monthly

*CD* cluster of differentiation; *FBC* full blood count; *IV* intravenous; *IVIG* intravenous immunoglobulins; *LFT* Liver function test; *M-ILD* Myositis associated interstitial lung disease; *PO* Orally; *TB* tuberculosis

### Corticosteroids

Corticosteroids, potent anti-inflammatories have formed the cornerstone of empirical treatment in chronic progressive M-ILD due to their rapidity of onset and relatively predictable side effect profile. Given their availability and ubiquity, they formed the standard of care for treatment of M-ILD even before the emergence of strong evidence supporting its usage [32]. A published meta-analysis demonstrated an efficacy (measured as functional improvement rate) of greater than 80% with use of corticosteroids alone in M-ILD, hence, advocating their use as monotherapy [33]. However, the 3 month survival rate of RP-ILD patients on corticosteroid monotherapy was 51.7%, lower than patients on other forms of immunosuppressive therapies and regime [33]. This possibly suggests that patients with RP-ILD are less responsive to corticosteroid monotherapy.

Prednisone, a widely prescribed oral corticosteroid may be administered at an initial dosing range of 0.5–1 mg/kg/day [34]. Alternatively, pulsed intravenous (IV) methylprednisolone may be given 1 g/day for 3 days [35]. As evidence suggest, additional immunosuppressive agents are often required for those patients who progress on steroid monotherapy [33]. Consideration should be given to their side effect profile, with regular glucose and bone density monitoring for those patients on long term steroids.

### Azathioprine

Azathioprine (AZA) an anti-metabolite, is one of the oldest immunosuppressive agents currently in use, exhibiting its effect primarily on inhibiting T-lymphocyte proliferation. It has been historically used as a steroid sparing agent [36]. However, there is a paucity of date regarding its use in connective tissue disease-ILD (CTD-ILD) and published data includes only case series and small uncontrolled clinical trials [37, 38]. Moreover, the data for M-ILD is meagre.

A retrospective study examined 66 patients who had received AZA as monotherapy in M-ILD over 60 months demonstrated that AZA was effective on the overall improvement of FVC, diffusion capacity of the lung for carbon monoxide (D_L_CO), and reduction of daily prednisolone dose [39]. Drug discontinuation rate was 17% of patients due to nausea and transaminitis, with other adverse events including opportunistic infections, haematological abnormalities, and non-melanomatous skin cancers [39]. While results appear encouraging, there was a low follow up rate at 60 months likely underpowering the study. Authors from another single centre retrospective study reported the use of AZA in patients with fibrotic CTD-ILD (including 15 patients in M-ILD) was associated with a statistically significant yearly improvement of FVC and D_L_CO (1.53%; 95% CI 0.19–2.87%; *p* = 0.025 and 4.9%; 95% CI 1.53–8.3%; *p* = 0.004 respectively) [40]. Results should be interpreted with caution as additional immunosuppressive agents were used in some patients, precluding accurate assessment of AZA as monotherapy.

### Mycophenolate mofetil

Mycophenolate mofetil (MMF), another anti-metabolite immunosuppressive agent that exerts its effects by suppressing the growth of B and T Lymphocytes via inhibition of inosine monophosphate dehydrogenase. It is well established as an immunosuppressive agent following organ transplantation as well as in some treatment of CTD especially scleroderma [41]. Similar to AZA, MMF is primarily used as a steroid sparing agent [36].

A retrospective study looking at the efficacy of MMF in CTD-ILD (including patients with PM/DM-ILD) demonstrated improvement in lung function up to 156 weeks (FVC, 7.3% ± 2.6%, *p* = 0.004; D_L_CO 7.1% ± 2.8%, *p* = 0.01) [42]. When MMF is directly compared to AZA in another retrospective study of patients with M-ILD, MMF improves the % FVC predicted and led to a reduction in daily prednisolone dose [39]. In this study, although there was no improvement in the % D_L_CO change in the MMF group unlike the patient group on AZA, MMF was better tolerated with less severe adverse events at (13.6% vs. 33.3%; P = 0.04) [39].

Whilst malignancy is a risk with immunosuppression, an observational cohort studies demonstrated no difference in low-level malignancy in CTD-ILD patient treated with either MMF or AZA compared to those not on treatment [43].

### Rituximab

Rituximab is a chimeric monoclonal antibody targeting the B cell surface marker CD20 + which results in B cell depletion. Its use has been well established in rheumatic conditions such as rheumatoid arthritis and it is increasingly used with success in CTD-ILD [44]. Its use as monotherapy in M-ILD is rare, and is often used as a steroid sparing agent in refractory cases [45].

An open-label, phase II trial involving 12 ASS patients with ILD manifestation reported that rituximab improves or stabilizes PFTs (FVC or D_L_CO) in the majority of patients at 18 month time point; improvement rate of PFT was 50% (95% CI, 19–81) [46]. The results of the trial was supported by several retrospective case series prior that reported that the use of rituximab in M-ILD led to the improvement in PFTs [47, 48]. Although the sample size of this study was small; results were promising and highlighted the need for larger clinical trial. More recently, the RECITAL trial released their phase 2b results where rituximab was compared to cyclophosphamide in CTD-ILD (including patients with M-ILD). The authors reported that participants in both the cyclophosphamide group and the rituximab group had increased FVC at 24 weeks (unadjusted mean improvement in FVC = 99 ml and 97 ml respectively), although one agent was not superior to the other [49]. On the other hand, an earlier observational retrospective study reported rituximab had better progression free survival compared to cyclophosphamide (CYC) at 2 years in patients with ASS related ILD [50].

### Cyclophosphamide

Cyclophosphamide (CYC) is an alkylating agent that is well established in the treatment of rheumatic disease. It was the most used immunosuppressive options in the treatment of scleroderma associated-ILD prior to the scleroderma lung study II, where MMF is now preferred due to its safety profile and tolerability [41].

Apart from the RECITAL trial where CYC was compared directly to rituximab, there are no randomized controlled trials examining for the use of CYC in M-ILD. The phase 2b RECITAL trial demonstrated that CYC was associated with an improvement in FVC (99 ml; SD 329), Global Disease Activity (GDA), Quality of Life (QoL) score at 24 weeks. Furthermore, there was a decrease in corticosteroid exposure up to 48 weeks [49]. In a small open-label study, 6 months of intravenous (IV) CYC in patients with DM/PM led to improvement in dyspnoea, > 10% improvement in FVC, and improvement in HRCT score [51]. The side effect profile from CYC included nausea, opportunistic infection, leukopenia, haemorrhagic cystitis [52].

### Calcineurin inhibitors

Conventionally, calcineurin inhibitors in M-ILD has included both tacrolimus and cyclosporin A and are frequently used following solid organ transplantation. The mechanism of action is by inhibiting interleukin-2-mediated CD4 + T-cell activation.

The first reported use of tacrolimus in M-ILD was in 1999 where it was used in a small cohort of patients who were refractory to conventional immunosuppressive therapy [53]. This was followed by a retrospective study involving 13 patients with anti-synthetase syndrome were treated with tacrolimus after they had failed to respond to conventional immunosuppressive therapy. The authors found that following a period of treatment with tacrolimus (twice daily dose at 0.075 mg/kg), all 12 patients demonstrated a statistically significant improvement in all three pulmonary variables (FVC, FEV1, and D_L_CO) with a statistically significant decrease in the requirement for corticosteroids [54]. Another retrospective study demonstrated in a cohort of 49 patients with M-ILD, tacrolimus was associated with long progression free survival and event free survival when compared to the steroid monotherapy, or in combination with cyclosporine or IV CYC [55]. The main side effects of tacrolimus are nephrotoxicity, hypertension, hypomagnesemia and tremors [56, 57]. At present, unlike the anti-metabolite agents i.e. AZA and MMF, experts maintain that tacrolimus is to be reserved for patients who progress either clinically or on follow up PFT despite being on combination of steroids and anti-metabolites [34].

Cyclosporin A (CsA) was first used in the treatment of M-ILD four decades ago suggesting initial efficacy in corticosteroid resistant disease [58]. Subsequent studies have shown that early initiation with CsA as part of an immunosuppressive regime with corticosteroids had a better survival outcome than those that had a delayed step-up approach [59]. Furthermore, in the same group of patients, there was stabilization of their ILD based on HRCT findings [59]. Nevertheless, CsA is not without its side effects in terms of hypertension, and gastrointestinal disorders hence, strict monitoring of plasma levels is required [57]. Appropriate levels have should be within 100–150 ng/ml at trough and 2 h post administration of and 1000 ng/ml to ensure an appropriate level of immunosuppression whilst also minimising the side effect profile [60].

More recently, tacrolimus has been compared directly to CsA by Fujisawa et al.in a prospective multicentre, open-label, randomized, 52 week phase 2 trial: prednisolone plus tacrolimus vs. prednisolone and CsA with the primary end point being progression free survival at 52 weeks. The authors concluded that combination treatment with prednisolone plus tacrolimus inferred better progression free survival [61].

### Anti-fibrotics

While immunosuppression is the hallmark of treatment in patients with M-ILD, there has been emerging evidence for the use of anti-fibrotics. A sub-analysis of the INBUILD trial looked at patients who had autoimmune disease related Progressive Fibrosing Interstitial Lung Diseases (PF-ILD) including those with CTD-ILD. In this study, nintedanib reduced the rate of decline in FVC compared to placebo over 52 weeks (− 75.9 ml/year with nintedanib vs − 178.6 ml/year; *p* = 0.012) [62]. As the trial was not designed specifically for M-ILD cases, there were very few patients with M-ILD recruited into this trial hence conclusion cannot be drawn with regards to the benefit of nintedanib in this group of patients. Nevertheless, the FDA have now granted approval for the use of nintedanib in PF-ILD cases, which includes patients with M-ILD. This is an important milestone as this will certainly open avenues for future prospective/retrospective trials investigating the efficacy of nintedanib in M-ILD. The most common side effects with nintedanib are gastro-intestinal related symptoms and transaminitis [62].

The other anti-fibrotic, pirfenidone was also assessed in RP-ILD patients in an open label study involving 27 patients. Although there was no observed overall survival benefit, the subgroup analysis of patients with subacute of ILD, had an improved survival compared to the historical groups alone (90% vs. 44%; *p* < 0.045) [63]. Side effects of pirfenidone include nausea, rash, and photosensitivity [64]. Given the evidence for shared fibrotic pathways in PF-ILD irrespective of the disease type, anti-fibrotic therapies may be promising therapeutic adjuncts in M-ILD [65, 66].

### Janus kinase inhibitor

Tofacitinib (TOF) is a Janus kinase (JAK) inhibitor that block multiples cytokines such as IL-6 and type I/II interferons; the former known to be elevated in PM/DM associated ILD [67, 68]. A case series by Japanese investigators demonstrated the potential use of TOF in RP-ILD. Five patients with anti-MDA5 positive antibody ILD were included, having failed combined therapy (corticosteroids, CYC, and CsA). Following institution of treatment, TOF conferred a survival advantage compared to a historical group of patients treated with combined immunosuppression alone without TOF [69]. Subsequently, Chen et al. describe the use of TOF in an open label prospective trial of 18 patients in patients with confirmed anti- MDA5 antibodies and ILD [70]. These patients received upfront TOF and corticosteroids with the majority of a patients having received no previous immunosuppression. Survival at 6 months was significantly greater in comparison to a historical group who did not receive TOF. There were also improvements in FVC, imaging and functional status over a period of time. These results can only be described as preliminary, with further larger case numbers required. The most common side effects from TOF are infections such as reactivation of CMZ, upper respiratory tract infections, bronchitis, and pneumonias [69, 71].

### Intravenous immunoglobulins

Intravenous Immunoglobulin (IVIG) has been used as treatment for various autoimmune conditions by clinicians for over 70 years due to its lack of immunosuppressive properties making it a favourable therapeutic choice [72]. Nonetheless, there are currently no prospective trials demonstrating its efficacy in treatment of M-ILD. A retrospective review of 17 patients with ASS previously on immunosuppression reported that over a period of two years, almost 40% of these patients had > 10% increase in their FVC after commencing on IVIG [73]. Moreover, these patients had previously not responded to other immunosuppressive therapies ad IVIG successfully reduced their steroid usage [73]. Otherwise, there have been various published single case reports demonstrating favourable outcomes with the use of IVIG as in both RP-ILD and refractory M-ILD [74–76]. Given its promising results despite limited data, IVIG may be added as salvage therapy in patients with refractory ILD given that it is not considered an immunosuppressive agent. IVIG therapy is generally well tolerated but rarely severe adverse events such as renal impairment, thrombosis, haemolytic anaemia, and transfusion-related acute lung injury (TRALI) may occur [77].

### Upfront combination therapy for RP-ILD

At present, most experts suggest up front aggressive multimodal immunosuppression i.e. combination of steroid therapy with one or two other agents particularly in cases of RP-ILD [78]. The British Society of Rheumatology recommends induction with high dose corticosteroids with immunosuppression to be used alongside in patients with RP-ILD. Recommended drugs that may be used as part of induction is rituximab or CYC, however tacrolimus and CsA may be considered as well [8]. Upfront combination therapy has been shown to improve patient outcomes in those with RP-ILD [13, 79]. Nakashima R et al. demonstrated that patients with RP-ILD on an intensive regime of prednisolone, CsA, and CYC had a 25-month survival rate of 75% vs. 28.6% compared to those who did not receive intensive regime. On the other hand, Matusudo KM et al. reported the combination of systemic corticosteroids, CYC and tacrolimus significantly improved FVC although there was no difference in mortality rate [79]. While evidence for induction combination therapy is still scanty at present, these studies demonstrate the potential role of combination therapy in RP-ILD. Clinicians however should be mindful of the possible synergistic side effects of combined immunosuppression with these agents.

### Non-pharmacological management

There are cohort of anti-MDA5 DM-ILD patients, who despite, early multimodal immunosuppressive agents, continue to progress. In these instances, interventional rescue therapies such as plasmapheresis, polymyxin B immobilised fibre may be indicated.

### Plasmapheresis

Plasmapheresis, also known as plasma exchange (PE), is an extracorporeal treatment employed to remove certain pathologic substances such as circulating autoantibodies, cytokines, immune complexes, endotoxins, and other substances from the plasma [80]. It has the ability to remove small to large size molecular weight particles by removing the entirety of the patient’s own plasma and replacing it with a healthy patient’s plasma. There are several case reports of its successful use in RP-ILD in CADM patients [81, 82] as salvage therapy. The exact timing of when to institute PE remains unclear but it should be considered as an emergent strategy in RP-ILD/severe ILD refractory to combined multimodal immunosuppressive therapy.

### Polymyxin B

Polymyxin B immobilised fibre column direct hemoperfusion (PMX-DHP) is an extracorporeal blood filter that absorbs harmful endotoxins. PMX-DHP was initially developed as a treatment for sepsis, but also has favourable effects on oxygenation in acute respiratory failure due to ARDS [83]. In cases of RP-ILD, it has been mainly used in the CADM with positive anti-MDA5 antibodies. There are several single case reports documenting some success of its use, where conventional immunosuppressive agents have failed patients [84, 85].

### Extra corporeal membrane oxygenation and lung transplant

Extra corporeal membrane oxygenation (ECMO) is a form of supportive care for patient with hypoxemia respiratory failure due to adult respiratory distress syndrome (ARDS), or occasionally, ILD. The optimal deployment strategy with regards to veno-veno or veno-arterial remains unclear, as is whether patients should receive concurrent mechanical ventilation or not. It can serve as a bridge to allow time for patients to respond to immunosuppressive regime, or while awaiting transplantation [86, 87].

Lung transplantation is a curative form of treatment however, in this cohort of patients, it is extremely challenging. Moreover, thorough transplant work-up is required in a patient who likely possesses various risk factors. Complications for a patient undergoing lung transplant include malignancy, myocarditis, and gastrointestinal complications such as dysmotility and aspiration risk, making the post-transplant period challenging, notwithstanding the profound myopathy pre and post transplantation [88]. A very recent multi-centre, retrospective study assessed the survival and prognostic factors in 64 lung transplant recipients with M-ILD, found that none of the patients experience ILD recurrence in the allograft and post-transplantation survival in M-ILD was similar to international all-cause-transplantation registries [89]. Crucially, patients with classical IIM (muscular involvement) have worse survival compared to than those who had amyopathic-IIM, however, the authors concluded that the association link found was not sufficiently strong to recommend a contraindication to lung transplant in classical IIM patients [89]. Thence, early lung transplant assessment and referral should be considered for the majority of these patients.

### Proposed algorithm

**Fig. 1:**
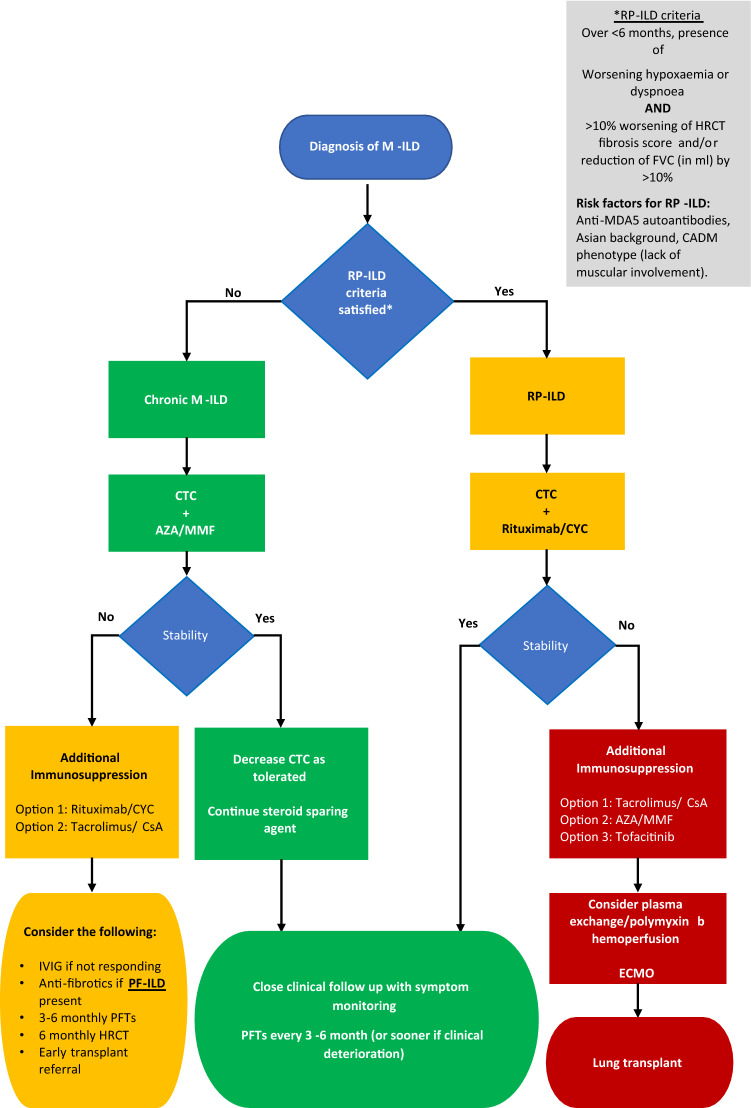
Proposed algorithm for the treatment of M-ILD. *AZA* Azathioprine; *CADM* Clinically amyopathic dermatomyositis; *CsA* Cyclosporin A; *CTC* Corticosteroids; *CYC* Cyclophosphamide; *ECMO* Extracorporeal membrane oxygenation; *FVC* Forced vital capacity; *HRCT* High-resolution computed tomography; *IVIG* Intravenous immunoglobulin; *M-ILD* Myositis associated interstitial lung disease; *MMF* Mycophenolate mofetil; *PF-ILD* Progressive fibrosing interstitial lung disease; *PFT* Pulmonary function test; *RP-ILD* Rapidly progressive interstitial lung disease

Based on current available evidence and our clinical experience in a combined respiratory and connective tissue disease specialist centre in Cork University Hospital, Cork, Ireland, we propose an algorithm to ease clinicians’ management decision in treating this heterogenous disease (Fig. 1). Dosing of each agent and recommended monitoring can be found in Table 3. Patients should ideally be managed in a specialist centre under the joint care of a rheumatologist and respiratory physician.

## Conclusion

Given the heterogeneity of presentation of M-ILD and the rarity of the disease process, there is a lack of robust evidence for the treatment or follow up for this group of patients. The initial choice of therapy should be guided by the mode of presentation, as well as the severity of the underlying ILD. Previous therapeutic algorithms and guidelines are based on expert opinions [16, 23]. Those who present with a RP-ILD require treatment with early aggressive multimodal immunosuppressive therapy such as high dose steroids, rituximab, CYC, and CNI with consideration of salvage agents, as well as interventional therapies such as plasma exchange, and ECMO if appropriate. Patients who present with milder more indolent disease trajectory, initial administration with steroids, and additional steroid sparing agents is appropriate, such as AZA or MMF. Future clinical trials powered specifically for CTD-ILD and M-ILD similar to the RECITAL trial, are urgently warranted to determine a robust evidence-based algorithm to aid clinicians’ management decision to treat this debilitating disease.

## Data Availability

No new data was created in this manuscript.
